# An image processing approach to computing distances between RNA secondary structures dot plots

**DOI:** 10.1186/1748-7188-4-4

**Published:** 2009-02-09

**Authors:** Tor Ivry, Shahar Michal, Assaf Avihoo, Guillermo Sapiro, Danny Barash

**Affiliations:** 1 Department of Computer Science, Ben-Gurion University, Beersheba, Israel; 2 Department of Electrical and Computer Engineering, University of Minnesota, Minneapolis, USA

## Abstract

**Background:**

Computing the distance between two RNA secondary structures can contribute in understanding the functional relationship between them. When used repeatedly, such a procedure may lead to finding a query RNA structure of interest in a database of structures. Several methods are available for computing distances between RNAs represented as strings or graphs, but none utilize the RNA representation with dot plots. Since dot plots are essentially digital images, there is a clear motivation to devise an algorithm for computing the distance between dot plots based on image processing methods.

**Results:**

We have developed a new metric dubbed 'DoPloCompare', which compares two RNA structures. The method is based on comparing dot plot diagrams that represent the secondary structures. When analyzing two diagrams and motivated by image processing, the distance is based on a combination of histogram correlations and a geometrical distance measure. We introduce, describe, and illustrate the procedure by two applications that utilize this metric on RNA sequences. The first application is the RNA design problem, where the goal is to find the nucleotide sequence for a given secondary structure. Examples where our proposed distance measure outperforms others are given. The second application locates peculiar point mutations that induce significant structural alternations relative to the wild type predicted secondary structure. The approach reported in the past to solve this problem was tested on several RNA sequences with known secondary structures to affirm their prediction, as well as on a data set of ribosomal pieces. These pieces were computationally cut from a ribosome for which an experimentally derived secondary structure is available, and on each piece the prediction conveys similarity to the experimental result. Our newly proposed distance measure shows benefit in this problem as well when compared to standard methods used for assessing the distance similarity between two RNA secondary structures.

**Conclusion:**

Inspired by image processing and the dot plot representation for RNA secondary structure, we have managed to provide a conceptually new and potentially beneficial metric for comparing two RNA secondary structures. We illustrated our approach on the RNA design problem, as well as on an application that utilizes the distance measure to detect conformational rearranging point mutations in an RNA sequence.

## Background

In the past several years, interesting novel RNA sequences were discovered that carry a diverse array of functionalities. By now, it is well known that RNAs are considerably involved in mediating the synthesis of proteins, regulating cellular activities, and exhibiting enzyme-like catalysis and post-transcriptional activities. In many of these cases, knowledge of the RNA secondary structure can be helpful in the understanding its functionality.

The importance of the secondary structure of RNAs presents a need for tools that rely on comparing two RNA secondary structures, which may indicate a functional commonality or divergence between them. These tools may usually accompany secondary structure prediction packages which are based on energy minimization such as Mfold [1] and the Vienna RNA package [2], both using the expanded energy rules [3] to predict the folding of RNA sequences. Calculating the distance between RNA structures have been approached by several methods, some of which are based on the edit distance of a tree representation of the RNA secondary structure elements [4-6]. An edit distance on homeomorphically irreducible trees (HITs) [7] was one of the original proposals for a comparison method. A different method was based on the alignment of a string representation of the secondary structures [8,9], where parenthesis represent the base-pairs, and another symbol represents unpaired nucleotides [6]. This representation is known as the dot-bracket representation. All aforementioned comparison methods were implemented as part of the Vienna RNA package [2,6]. More recent suggestions for RNA secondary structure comparisons include the use of context free grammars [10], alignment by dynamic programming [11], and a more general edit distance under various score schemes [12,13]. A method for a rapid similarity analysis using the Lempel-Ziv algorithm was suggested in [14]. Another method uses the second eigenvalue of the tree graph representation for the structures comparison, [15], and was later integrated into the RNAMute [16], a Java tool, which we will use for our second application illustration. The latter aforementioned method is not a metric. A comparison on metric methods is available in [17], where it was found that simple metrics work sufficiently well for measuring RNA secondary structure conservation.

Here, we propose an alternative distance measure, motivated by image processing and pattern recognition. The new metric is based on an analysis of the dot plot diagrams of the secondary structures, and uses histogram based correlation and plane group distance to calculate the similarity between the diagrams. The measure combines both fine and coarse elements in the structure and can offer an alternative method to the aforementioned distance measures, with a critical advantage in applications that use energy and probability dot plots for the analysis of secondary structures.

The idea of using two dimensional plots in order to investigate possible secondary structure elements in RNAs (these 2D plots in time became known as dot plots) can be traced back to a seminal work by Tinoco et al. [18]. In Trifonov and Bolshoi [19], such 2D plots have been used by their analysis to reveal common hairpins in 5S rRNA molecules. Jacobson and Zuker [20] later used dot plots to predict well defined areas in a viral genome, suggesting that the amount of cluttering in dot plots reflect the impossibility of accurate structure predictions. Horesh et al. [21] performed clustering into RNA families based on dot plots. The above works represent a variety of uses for dot plots when analyzing RNA secondary structures.

Our new distance measure will be examined in two application problems that require the use of distances between RNA secondary structures. The first is the RNA design problem, also known as the inverse RNA folding problem. The goal in this problem is to design nucleotide sequences that fold to a given RNA secondary structure. The design problem can be applied to noncoding RNAs, which are involved in gene regulation, chromosome replication, RNA modification [22], and other important processes. Various heuristic local search strategies have been used by existing programs dealing with inverse RNA folding. The original approach to inverse RNA folding was implemented in RNAinverse, available as part of the Vienna RNA package [6]. There, two different criteria were used to find the local optima: 1. mfe-mode: a structural distance between the mfe structure of the designed sequence and the target structure. 2. probability-mode: the probability of folding into the target structure. A second algorithm is called RNA-SSD (RNA Secondary Structure Designer) and was developed by Andronescu et al. [23]. It tries to minimize a structure distance via recursive stochastic local search. A recent algorithm that was devised to solve the design problem, called Info-RNA, can be found in Busch and Backofen [24].

The second application problem for illustrating our proposed distance measure is to predict mutations that cause a conformational rearrangement. Certain RNA molecules can act as conformational switches, by alternating between two states, and thereby changing their functionality [25-29]. RNA conformational switching was found to be involved in cell processes such as mRNA transcription, translation, splicing, synthesis and regulation. The conformational switching can be induced by a point mutation as well [30]. Given a thermodynamically stable RNA structure, we can try to predict a conformational rearranging point mutation by traversing all possible single point mutations of a sequence and locate the most significant ones, in terms of secondary structure difference [31]. RNAMute [16] and RDMAS [32] are tools that attempt to perform such predictions and are based on energy minimization methods [1,2]. The RNAMute mutation analysis tool, [16], includes RNAdistance from [2,6]: the RNA edit distance of the dot bracket representation as a fine-grain comparison method, and the edit distance of the Shapiro representation, [4,5], as a coarse-grain comparison method.

We have developed a stand-alone procedure called DoPloCompare, which receives two RNA structures as an input, and calculates their similarity grade using our new distance measure algorithm. In order to illustrate our metric, we have constructed several test cases that use the DoPloCompare procedure for the distance measure, in the framework of the two applications described above.

In the following sections we will describe the new procedure DoPloCompare, the two application problems we use for illustration, and the results obtained when testing DoPloCompare on these applications. We discuss its contribution alongside commonly used routines such as RNAdistance [6].

### DoPloCompare – comparing two RNA secondary structures

The basis for our algorithm is the fact that a base-pairing indicator dot plot diagram is a sound representation of the RNA secondary structure, as will be detailed in the next Section. In general, a dot plot is a matrix comparison of two sequences (or one with itself) and is prepared by sliding a window of user-defined size along both sequences. If the two sequences within that window match with a precision set by the mismatch limit, a dot is placed in the middle of the window signifying a match [33]. In the case of RNA sequences, we assume that a similarity between dot plot diagrams of two sequences is a good criterion for similarity between the secondary structures of those sequences.

**Figure 1:**
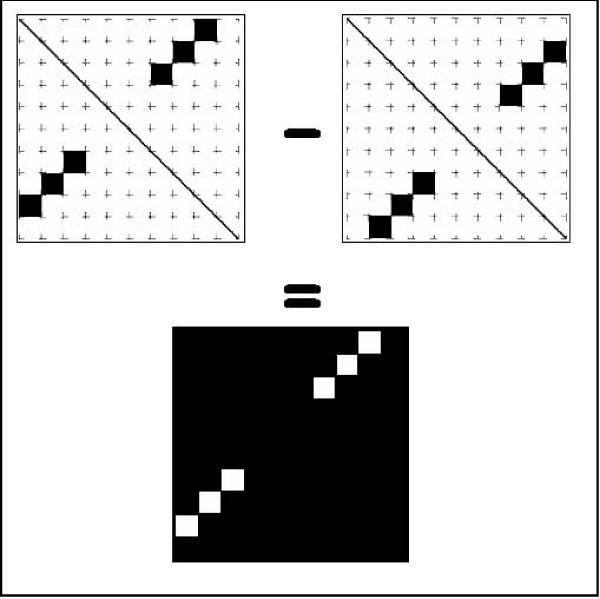
**Dot plot subtraction**. The test case demonstrating the effect of image subtraction for measuring the distance between two dot plots shows a non-desirable result. Although containing a similar secondary structure, the subtraction of the right dot plot from the left dot plot yields a high number of pixels in the resultant image, which translates to a large distance instead of the desired zero distance. At best, when a cut-off for the intensity of one of the secondary structures is used when subtracted from the other, we remain with the pixels belonging to the other structure that appears intact.

Given two dot plot diagrams of two secondary structures, we would like to develop a distance grade that best indicates how well the secondary structures attached to the diagrams resemble each other. When two structures are similar, we require the distance between their representing dot plot diagrams to be small (discarding "simple" image subtraction as a non-desirable option, as can be observed in Figure 1), and alternatively, when the structures are different, we require that the distance will increase.

#### Observations

Two main observations served as motivation in establishing the distance calculation formula. The first is that similar secondary structures will maintain matching dot plot diagrams with dots in the same or in close positions. Obviously, two secondary structures will look alike if all or most of the base pairing couples will be located in the same or in proximal places in the sequences. The second observation is that two secondary structures will count as similar if both the number and order of the elements they contain are the same [15]. For example, two RNA structures with four stems can be considerably different if the first structure is arranged as a one elongated structure containing a bulge and three consecutive loops, while the second includes a bulge, a multi-branch loop, and two additional stem-loops that branch out of the multi-branch loop. From the second observation, we concluded that the calculation should also reflect the overall arrangement of elements in the secondary structure, and the groups of points in the dot plot diagrams accordingly. All these observations raise the motivation to compare dot plots, by considering them as simple images and exploiting tools from image processing.

#### Distance calculation

Taking into account the above observations, we have developed the following distance grade formula.

Let *O *be the dot plot diagram of the original sequence representing its secondary structure.

Let *M *be the dot plot diagram of the mutated sequence representing its secondary structure.

Then:

(1) $$Distance\text{\_}Grade(O,M)=\frac{Dist(O,M)}{Corr(O,M)}$$

Where *Corr *stands for Correlation and *Dist *stands for Distance. For the Correlation part we used the histograms method as detailed in the Methods Section. In our implementation, we used a 4-dimensional histograms correlation:

(2) $$\begin{array}{c} Corr(O,M)=\\ \sqrt{Xc(O,M)\times Yc(O,M)\times Dc(O,M)\times Ic(O,M)} \end{array}$$

Where:

• Xc(O, M) is the correlation grade (see Equation 4 in Methods) between the vectors that sums all the points on each X column of the matrix

• Yc(O, M) is the correlation grade between the vectors that sums all the points on each Y row of the matrix

• Dc(O, M) is the correlation grade between the vectors that sums all the points on each Diagonal SW-NE

• Ic(O, M) is the correlation grade between the vectors that sums all the points on each Inverse Diagonal SE-NW

For the distance (Dist) part we used the RMS distance as explained in the Methods Section, and applied it on the groups of points of both dot plot diagrams. Note that in case the correlation value is zero, the formula will return an infinity value. There is no practical interest in this case, since it is only possible when at least one of the dot plot diagrams represents a trivial structure of a single stranded RNA, which has no biological significance from a structural standpoint. For safety from the numerical standpoint, if encountering a zero correlation value, our system returns the distance (Dist) grade alone in this situation.

#### Formulas explanation

The histogram correlation (*Corr*) compares the locations of every *p*_*i *_and *p*_*j *_under the best matching shift, where *p*_*i *_is a pixel in the original sequence's dot plot diagram, and *p*_*j *_is a pixel in the mutated sequence's dot plot diagram. However, in some cases small differences in the locations of the pixels between the original and the mutated dot plot diagrams, reduces the correlation grade. Literally, the grade is reduced for every pixel in the original dot plot that is not placed on the same exact location as a pixel in the mutated dot plot. For this reason, we introduce a distance measure between the dot plot diagrams, in addition to the histogram correlation.

The histograms formula is well balanced between all the different vectors being correlated: First, the Xc and Yc vectors represent the base pairing arrangement along the sequence. Note that the dot plot diagrams described in this article are symmetric matrices, thus both Xc and Yc vectors are exactly the same (non symmetric diagrams are described in the Dot Plot Diagrams Subsection in the Methods Section). Future extensions might utilize non-symmetric diagrams, and will be supported by our system.

Second, the Dc vector describes the long stems arrangement in the structure. Finally, the Ic vector corresponds to the projection of the overall structural elements arrangement. This combination allows the formula to be tolerant to small structural differences. For example, when comparing two long stems, distinguished by a single bulge in the middle, the Dc vectors will be very different between these two structures, but the other three vectors will remain similar, thus the correlation grade will remain high. The distance measure (*Dist*) is more tolerant to small differences and represent overall proximity between the sets of points. Moreover, if a pixel in the original dot plot is not placed on top of a pixel in the compared dot plot, the correlation grade will be reduced equally, regardless of the distance between the pixels, while the distance measure will be reduced in a direct proportion to the distance between the pixels.

#### Illustration

**Figure 2:**
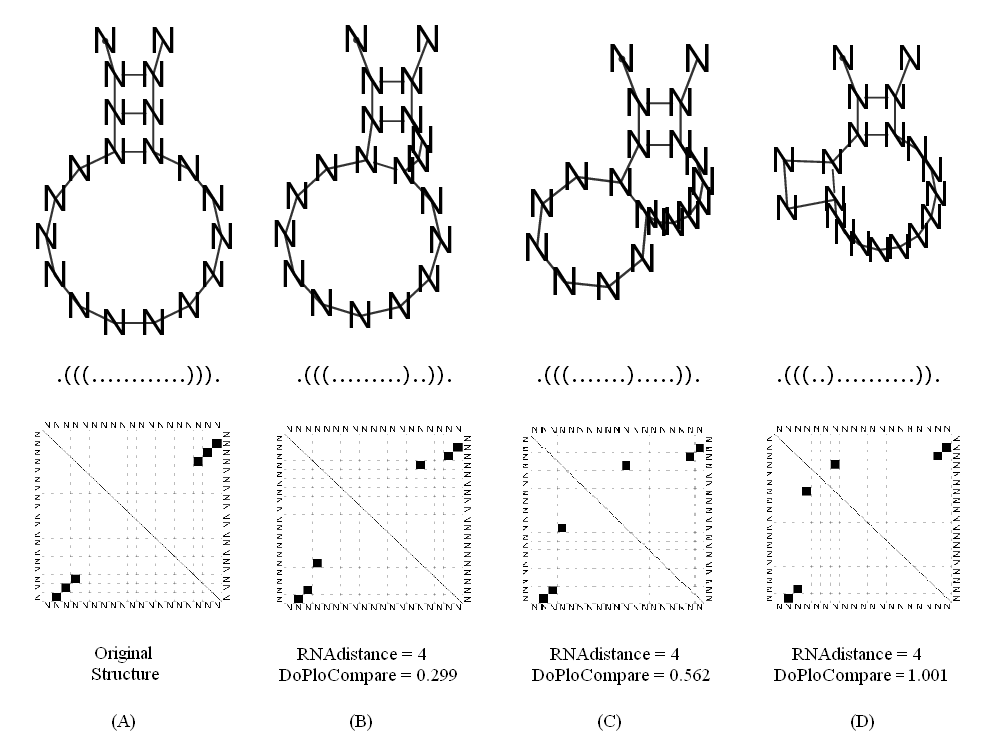
**Illustration of the difference between RNAdistance and DoPloCompare**. An example of the difference between RNAdistance and DoPloCompare, illustrated on three structure comparisons. In each case, the compared structure appears next to its representing dot-plot diagram and its dot-bracket notation. The comparisons are relative to the original structure depicted in (A). While RNAdistance = 4 remains the same in (B), (C), and (D), DotPloCompare values increase as the structure visually diverts from the original structure.

The distance grade will be high in the following cases: when the correlation value is low and/or when the distance value is high. A low correlation value will be calculated when the compared diagrams' vectors are distinct. A high distance value will be calculated when the compared diagrams' groups are distant – see the example in Figure 2. From these comparisons, we argue that there is an advantage in using our DoPloCompare over RNAdistance since structure (D) in the Figure is more remote from structure (A) than structure (B) or (C) as DoPloCompare values indicate.

#### DoPloCompare program flow

DoPloCompare receives two RNA secondary structures as input, either in a dot bracket notation or as two ct files (produced by Mfold [1]). The main flow of the algorithm is made of three parts:

1. Build the dot plot matrix from the secondary structures.

2. Compare the two structures using formula (1) for the distance grade. In order to normalize the distance grade, it is divided by the length of the sequences.

3. Output the distance grade.

#### Building the Dot Plot Matrix

Taking the simple matrix characteristics (described in the Methods Section), one can easily build such a matrix by traversing a folding option received as an output of any folding program, and for every base pairing nucleotides couple in the sequence set the matching matrix cell value to 1 (other cell values will be set to 0).

### Incorporating DoPloCompare into RNAinverse

As part of RNAinverse (see RNA-Design Subsection under the Methods Section) operation, it uses a distance score to measure the designed sequence's structure to the input (the desired secondary structure). When the distance between the input and the structure is zero, the operation ends and the application outputs the sequence. In some cases, the input structure is undesignable, i.e. in these cases the secondary structure of the input is not energetically favorable and it is impossible for the algorithm to predict a sequence with the same secondary structure as the input structure. In this case the algorithm finds a close match based on the structural difference, i.e. a sequence and a structure with the smallest distance from the input structure.

We have replaced the base pairing distance measure used by the RNAinverse algorithm with DoPloCompare, thus creating a new version of the algorithm for the RNA design problem that is based on our image processing distance proposed instead of base pairing distance.

### Finding the most significant point mutation using DoPloCompare

The system is based on both histograms and geometry as the core comparing mechanism between the original sequence secondary structure and all the possible point mutations' folding variants. The algorithm is composed of two major parts: pre-processing and main comparing mechanism. The pseudo-code of the algorithm is given here:

Most_Significant_Mutation ( Original_Sequence )

BEGIN

   Original_Matrix:= Built matrix

      from the folding of Original_Sequence;

   Max_Grade:=0;

   Max_Sequence:=Original_Sequence;

   WHILE ( Mutated_Sequence:= Next

      point mutation of Original_Sequence )

   BEGIN

   Mutated_Matrix:=Built matrix from the

         folding of Mutated_Sequence;

      Grade:=Distance grade between

         Original_Matrix and Mutated_Matrix;

      If ( Grade > Max_Grade )

      BEGIN

         Max_Grade:=Grade;

         Max_Sequence:=Mutated_Sequence;

      END

   END

   Return Max_Sequence;

END.

#### System parameters

The system has several parameters, including:

• Folding program – either MFOLD or Vienna's RNAsubopt.

• Number of suboptimal folding options to be considered by the algorithm.

• Geometric distance measure to be used – either RMS or Hausdorff [34] distances. The default measure is RMS.

#### Pre-processing

The pre-processing part is divided to three steps (each is described in detail in the Methods Section):

1. Create all single-point-mutations in the original sequence.

2. Fold the mutated sequences using the folding program of choice.

3. From the folding program's output, we build a dot plot like matrix.

#### Main comparing mechanism

The mutated and original secondary structures' representing dot plot matrices are being compared using the DoPloCompare application (see 'DoPloCompare' section). Each mutated sequence's dot plot matrix receives a distance grade, which represents its similarity to the original sequence's representing matrix.

#### Output

At this stage, the algorithm finds the dot plot with the highest distance grade, i.e., the dot plot with the greatest difference from the dot plot diagram of the original sequence. This dot plot represents the secondary structure of one of the suboptimal folding options of a mutated sequence. The algorithm reports this sequence, along with additional data:

1. A representation of the secondary structure – either a dot-bracket in the case of RNAsubopt or a ct file in the case of Mfold.

2. The location of the point mutation and the replaced nucleotide (e.g., G15U).

3. The dot-plot-like matrix of the mutated sequence.

In addition, for user convenience, the secondary structure and the dot-plot-like matrix elements of the original sequence are also attached.

## Results

### The RNA-design problem

**Figure 3:**
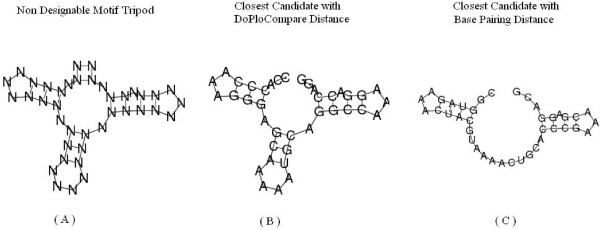
**Structural element tripod showing success for DoPloCompare**. The structural element Tripod [24]. (A) The desired secondary structure for which the algorithm tries to design a sequence, the element is composed of four stems, three of which with terminal hairpin, surrounding a multibranch loop. (B) The exact solution found when using the modified RNAinverse with DoPloCompare distance. (C) The closest secondary structure the algorithm returns after 500 iterations, when using the original RNAinverse with base-pairing distance.

**Figure 4:**
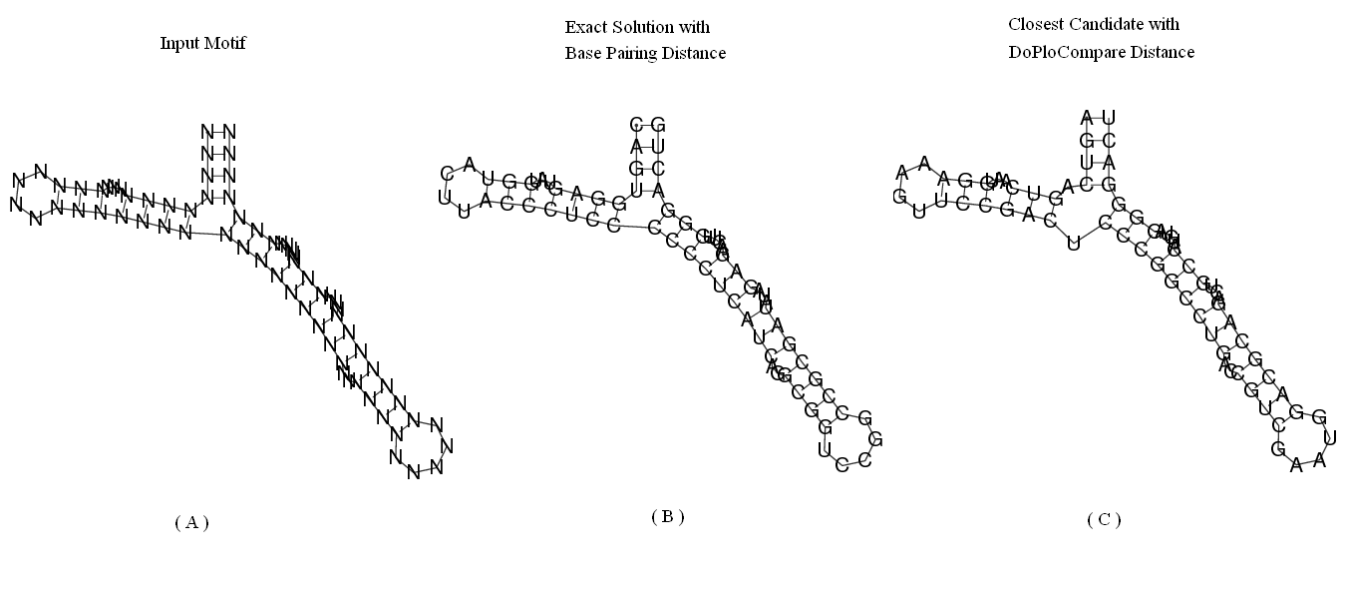
**Generated case showing success for base-pairing distance**. Structural element taken from a generated set of secondary structures with uniform probability. (A) The desired secondary structure for which the algorithm tries to design a sequence. (B) The exact solution found when using the RNAinverse with base-pairing distance. (C) The closest secondary structure DoPloCompare returns after 500 iterations.

**Figure 5:**
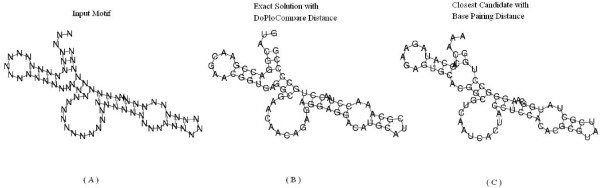
**Generated case showing success for DoPloCompare**. Structural element taken from a generated set of secondary structures with uniform probability. (A) The desired secondary structure for which the algorithm tries to design a sequence. (B) The exact solution found when using the modified RNAinverse with DoPloCompare distance. (C) The closest secondary structure the algorithm returns after 500 iterations, when using the original RNAinverse with base-pairing distance.

We have compared the results of RNAinverse using DoPloCompare vs. the results when using a base pairing distance. As explained above, RNAinverse deals with two types of input structures, designable and undesignable. In the designable case there is no advantage for either one of the approaches, both produce sequences that fold into the given secondary structure. This is due to the fact that identical structures lead to zero distance in both distance measures. Table 1 presents an example of five designable structures. However, for the undesignable case when using RNAinverse with base pairing distance (the first taken from [24]), we found several examples where DoPloCompare is able to reach an exact answer. For fairness, there are also examples where RNAinverse reaches an exact answer and DoPloCompare does not, within 500 iterations. Three example secondary structures are depicted in Figures 3, 4, 5 for illustration. The first structure is called Structural-Element-Tripod and describes a tripod like structure with three hairpins surrounding a multibranch loop, found in [24]. It shows a case that was noted before in the literature in which RNAinverse is not able to provide an exact answer whereas DoPloCompare does reach an exact solution. The second and third cases, respectively, are taken from the generated sample explained in the next Section. The second case is a one in which RNAinverse succeeds to reach an exact solution whereas DoPloCompare does not, and the third case is similar to the first case by illustrating once again a success for DoPloCompare while RNAinverse fails. For all three test cases we executed 500 runs and the Figures present: (a) the given structure; (b) an exact solution found with DoPloCompare or base pairing distance, respectively; (c) the best result achieved when using base pairing distance or DoPloCompare, respectively.

**Table 1:** Designable RNA secondary structures

Index	Structure in dot-brackets notation	Length (nt.)	Output sequence of original RNAinverse [6]	Output sequence of modified RNAinverse [6]
1	(((...(((...(((...)))...(((...)))...)))...)))	45	ACCGCCAGACAGGGCCAAGCCA-	UCCAAAUUCAUAGUAUAAUACA-
			CAUCCUAAGGAAAAGAAAAAGGA	CAUCCUAAGGAAAAGAAAAAGGA

2	(((.((.(((..((((((((.......)))))))))))...)).)))	47	GCUGUAGCCAAGUGGUAGUUGCU-	GCGUUCCGUCAGACUCAUGAGGC-
			AUAAAAUUAUUAUGGAUGUAGGGU	UAGGUCAUGGGUGACUCAGACCGC

3	(((((..((....((((.....)))).....))...)))))	41	GUCUGAAGCUCAAUGAUCUC-	CUAGACCUCUUUAGUGGAAC-
			CAAUUAAACUCGUGUACGGAU	GGCCGCGGACUGAAUAUCUAG

4	.((((((((((((...((.......))...))))))))).)))	43	CUCGUGAAUAUAACACUCAAG-	AUUCAAAAAAAACAAAUCAAA-
			GACCGAAAUUUAUGUUUAGUGA	AAAAGAAAAGUUUUUUUUAGAA

5	((((..(((...((...(((((....)))))...))...)))))))	46	GGUUCAGUUCAUUGCUCAUACUU-	UAUGUUAUCAAUUGUUGGCAUGC-
			AACGGUAUUCUCGUACGACAACC	AGUCAUGCAUUCAUAGGGUCGUG

This table displays the results for five designable secondary structures, comparing the original RNAinverse (using base pairing distance) output sequence, with the modified RNAinverse (using our proposed DoPloCompare distance) output sequence. Both algorithms succeeded in designing a sequence that folds into the input secondary structures, depicted in the second column, thus providing sequences with zero distance to the input structure.

#### Statistical comparison

Stochastic methods are needed in order to solve the RNA inverse folding problem. Therefore, a statistical comparison on an unbiased set is required when evaluating the merits of the new distance measure for providing a better solution to the design problem. In order to generate a set of secondary structures with uniform probability, the program ranstruc [35] that was kindly given to us by the authors of this reference was used.

Without loss of generality, we first chose a minimum stem length of 7 nt, generated 1000 random structures, and compared the performance of both programs with a fixed number of starting points, 1000 each. We ran this procedure for sequences of three different lengths: 70, 100, and 150 nt. For 70 nt, 150 iterations of RNAinverse and DoPloCompare were used and all structures were designable. For 100 nt, 300 iterations of RNAinverse and DoPloCompare were used and 2 structures were found undesignable. For 150 nt, 700 iterations of RNAinverse and DoPloCompare were used and 3 structures were found undesignable. There was no advantage or disadvantage to using DoPloCompare over the standard RNAinverse and vice versa. Next, we chose a minimum stem length of 3 nt, for the length of 50 nt. Out of 10,000 structures generated by the progam ranstruct, 40 structures were more difficult to design with a low number of iterations, but with 500 iterations, both DoPloCompare and RNAinverse with base pairing distance were able to solve them exactly. In order to find more difficult cases that are undesignable, we generated structures of length 70 nt. Out of 500 structures generated, 15 structures were impossible to design for either one of the distances while the other was able to find an exact solution. Two of these examples are depicted in Figures 4 and 5.

These results show that RNAinverse with the integrated DoPloCompare distance grade is able to outperform the original RNAinverse that utilizes the base pairing distance in some cases, while in others the opposite occur. The statistical comparison shows that there is no clear-cut advantage to either one of the distances but there are cases in which one method fails while the other succeeds.

### Finding the most significant point mutation

We compared the three test cases that were used in [15] before and after inserting DoPloCompare. Additionally, we tested our system on a data set of ribosomal RNA pieces (the sequence for each piece is available in Additional file 1.

#### Wild type sequences

We will describe the results for three well-studied RNA sequences that were used in [15] for a bioinformatics proof of concept. It is worthwhile noting that we are looking for the mutation with the largest structural difference from the wild type, while in [15] the ultimate goal was to look for a mutation that can lead to a bistable conformation. We successfully locate mutations that lead to a folding rearrangement with large difference from the wild type structure, and that are similar to the ones found in [15]. In addition to the second eigenvalue classification, we specifically compare our results to RNAdistance's dot bracket edit distance grade, which was mentioned but not directly used for comparison in [15]. RNAdistance was later integrated into RNAMute [16].

#### Leptomonas collosoma

**Figure 6:**
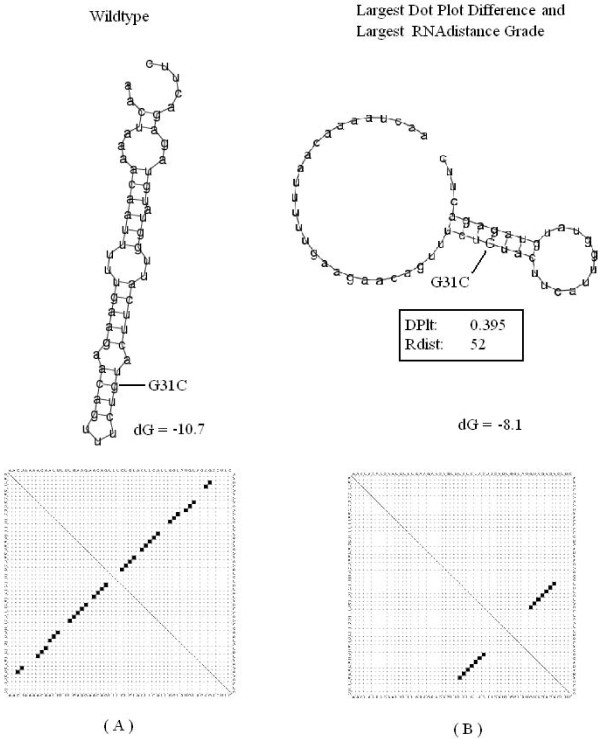
**L. collosoma**. The predicted most significant mutation for the spliced leader RNA from *L. collosoma*. (A) Wild-type folded structure along with its representing dot plot matrix. The computed RNAfold global minimum energy is dG = -10.7. (B) The mutated folded structure with the largest distance grade from DoPloCompare (DP) = 0.102. The largest RNAdistance grade was also recorded for this structure (Rdist) = 52. The computed RNAfold global minimum energy is dG = -8.1 kcals/mole.

The first sequence is the spliced leader RNA from *Leptomonas collosoma *which was studied by LeCuyer and Crothers [30,36], where they experimentally demonstrated a mutation induced RNA switch. In this test case, our system reported a structure with one double strand segment and a hairpin. This structure is of larger difference from the optimal wild type folding than the one reported in [15] that contains a bulge and a hairpin. We assume that this difference emerges from the different folding parameters, because the second eigenvalue of our result is also 1.0 (see [15]). A supporting fact for the latter is that when taking the largest RNAdistance grade, we obtain the same mutation and suboptimal folding as ours. The results are presented in Figure 6.

#### P5abc subdomain

**Figure 7:**
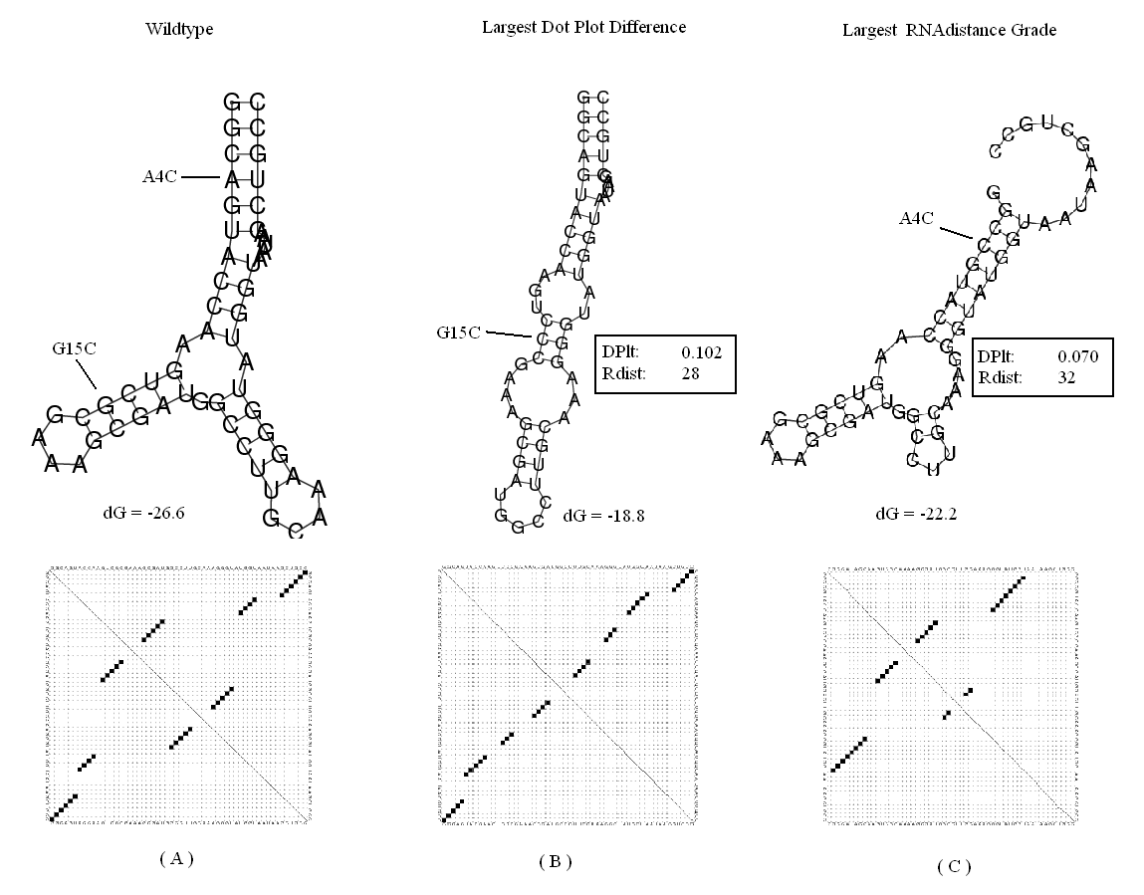
**P5abc subdomain**. The predicted most significant mutation for the P5abc subdomain in the group I intron ribozyme of the *T. thermophila*. (A) Wild-type folded structure along with its representing dot plot matrix. The computed RNAfold global minimum energy is dG = -26.6. (B) The mutated folded structure with the largest distance grade from DoPloCompare (DP) = 0.102. The RNAdistance grade for this structure (Rdist) = 28. The computed RNAfold global minimum energy is dG = -18.8. (C) The mutated folded structure with the largest RNAdistance grade (Rdist) = 32. The DoPloCompare grade (DP) = 0.070. The computed RNAfold global minimum energy is dG = -22.2 kcals/mole.

The second sequence is the P5abc subdomain of the *tetrahymena thermophila *ribozyme that was studied by Wu and Tinoco [37]. The results for the second sequence are found in Figure 7. In this test case, our system predicted the mutation G15C, which was also reported in [15] as a solution. When testing the P5abc subdomain with Mfold, both G15C and G15U produced the same dot plot matrix in one of their suboptimal folding options, thus receiving the same similarity grade. The mutation C22G produced a very similar matrix, with a somewhat lower similarity grade. In this case, the largest RNAdistance grade was received in the mutated structure of A4C, which is more similar to the original structure than our results. Both the A4C mutation and the original structure contain a multi-branch loop, while our reported mutation's structure does not.

#### Hepatitis delta virus

**Figure 8:**
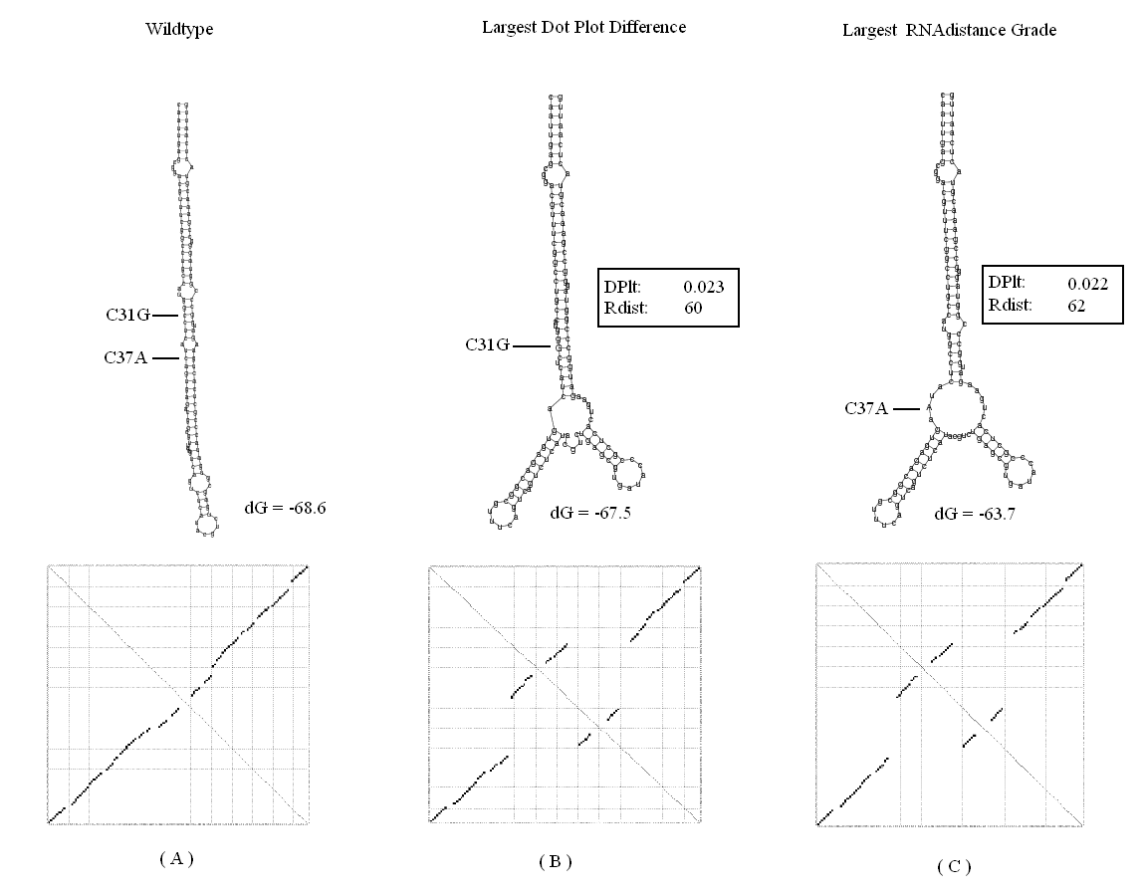
**Delta virusoid**. The predicted most significant mutation for the virusoid sequence from Hepatitis delta virus. (A) Wild-type folded structure along with its representing dot plot matrix. The computed RNAfold global minimum energy is dG = -68.6. (B) The mutated folded structure with the largest distance grade from DoPloCompare (DP) = 0.023. The RNAdistance grade for this structure (Rdist) = 60. The computed RNAfold global minimum energy is dG = -67.5. (C) The mutated folded structure with the largest RNAdistance grade (Rdist) = 62. The DoPloCompare grade (DP) = 0.022. The computed RNAfold global minimum energy is dG = -63.7 kcals/mole.

The third sequence is taken from human hepatitis delta virus ribozyme that was studied by Lazinski et al. [38], for its regulation of self-cleavage activity. The results for the third sequence are found in Figure 8. In this test case, our system predicted the C31G mutation. The structure induced by this mutation is similar to the one in [15]. The U40G that was suggested in their research [38] maintained a similarity grade that was very close to the grade of our system result. In [38], the authors mention the existence of eight possible mutations that provide the desired non-linear effect in the ribozyme structure, and this may explain the variation. The largest RNAdistance score was recorded in a highly similar structure to the one found by our system.

#### Ribosomal data-set

We have generated a data set of small RNA sequences, containing fragments that were cut from the rRNA of the *thermus thermophilus *[39]. This data set was built in order to test our system and compare its results to the RNAdistance results. Labels for the data set can be found in Additional file 1.

**Figure 9:**
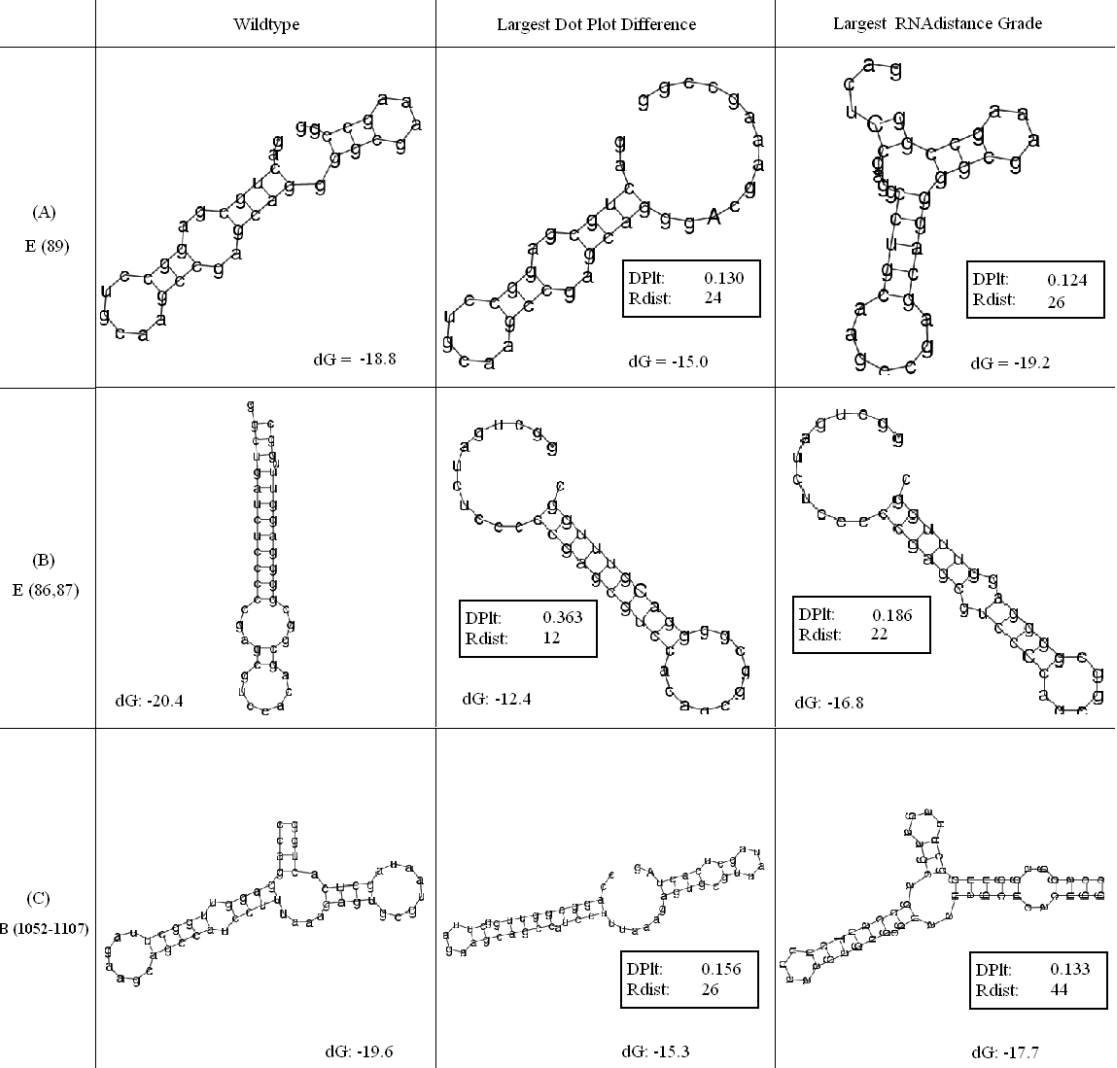
**Ribosomal data-set differences**. Three examples from the ribosomal data set that produced differences between our system proposed structure and the structure with the largest RNAdistance. (A) The original structure of item *E*_(89) from the ribosomal data set (left) along with our system resulted structure (center) and the structure with the largest RNAdistance (right). (B) The same results set for *E*_(86, 87). (C) The results set for *B*_(1052 – 1107).

Out of the 21 RNA sequences in the data set, 16 produced the same exact mutation and structure as the ones received by comparing the edit distance of the dot bracket representation of the folded structures. Two sequences produced different mutations but highly similar structures to the results from RNAdistance. Regarding the remaining three sequences, there were differences between our system's result and the largest RNAdistance result:

1. Our proposed structure for the *E*_(89) is different than the structure with the largest RNAdistance, but it is non-obvious to determine which one of them is more significant, both of the mutations alter the structure with respect to the original structure, as observed in Figure 9(A).

2. Our proposed structure for the *E*_(86, 87) is quite similar to the structure with the largest RNAdistance. However, both the RNAdistance structure and the original structure contains an extra loop. Thus, it can be argued that our proposed structure is less similar to the original one, as observed in Figure 9(B).

3. Our proposed structure for the B_(1052–1107) is less similar to the original structure than the structure with the largest RNAdistance. Both the original and RNAdistance's structures contain a branch that is not present in our system's result, as can be observed in Figure 9(C).

The ribosomal data set results are summarized in Table 2. Labelings for the sequences that are used in Table 2 are reported in Additional file 1.

**Table 2:** Ribosomal data-set

Index in the data set	Sequence name	Length (nt.)	Our predicted mutation	Mutation with largest RNAdistance [6]
1	A_(765–816)	52	**G7C**	G7C
2	E_(68)	46	**C28G**	C28G
3	A_(1241–1296)	56	G33C^(*A*)^	G32C
4	A_(820–879)	53	**C4A**	C4A
5	A_(588–651)	64	**G38C**	G38C
6	A_(995–1045)	55	**G41C**	G41C
7	B_(1052–1107)	56	G55A^(*B*)^	C28U
8	B_(589–668)	82	**G37U**	G37U
9	A_(136–227)	93	**G10U**	G10U
10	A_(1113–1187)	74	**G60U**	G60U
11	B_(865–911)	46	**C38G**	C38G
12	E_(2676–2731)	57	**C3A**	C3A
13	E_(99,100,101)	79	**G9C**	G9C
14	E_(90,91,92)	76	G44A^(*A*)^	G43A
15	E_(89)	43	G36C^(*B*)^	A23C
16	D_(8,9,10)	53	**C36G**	G31U
17	A_(1420–1480)	56	**G47C**	G47C
18	A_(240–286)	47	**U5C**	U5C
19	A_(442–492)	41	**G24U**	G24U
20	E_(65,66)	57	**U22A**	U22A
21	E_(86,87)	39	G29A^(*B*)^	G5C

This table summarizes the results for the ribosomal data set, comparing our system results to the results with the largest RNAdistances. In the fourth column we present our system's predicted mutation. When the resulted mutations are identical to RNAdistance, they are presented in bold face. (A) Marks the 2 sequences with a different mutation but similar structure. (B) Marks the 3 sequences with different secondary structure (refer also to Figure 8 and Additional file 1).

## Discussion and future work

We have described a method to compare two RNA secondary structures, and to assign a grade to this comparison based on the similarity of their representing dot matrices. This measure is different than the known measures by the fact that it compares geometrical and planar distances between dot plots that represent structures as opposed to traditional base pairing or edit distance methods between trees or graphs that represent structures. In order to compare this novel measure and considering its unique characteristics, we first showed its advantage on a synthetic case and then illustrated it in two applications that use this measure as the core distance mechanism. In the first application, the RNAinverse, we have shown that our method is capable of outperforming in several cases the traditional base pairing distance for the undesignable input structures. In the second application, we have adopted this method to predict the most significant point mutation for a given sequence in terms of its structural effect on the wildtype, and provided interesting results in comparison to other known methods. We have compared our application results to the commonly used RNAdistance module that is part of the Vienna RNA package [2,6], and the classification by the second eigenvalue that was provided for three example test cases in [15]; the first result, from *Leptomonas collosoma*, was less similar to the original structure than the one predicted in [15] (i.e., in this test case our system surpassed). For the second result, the P5abc subdomain, our system predicted a mutation that was proposed in [15], and on the final result, from the hepatitis delta virusoid, we have predicted a very similar structure to the one found by the second eigenvalue method. Overall our system matched or even outperformed the second eigenvalue method results. Concerning the results for the ribosomal data set, which were compared to RNAdistance's results: the results were identical in 16 out of the 21 RNA sequences, two sequences produced different mutations but highly similar structures to the results from RNAdistance, and for the remaining three sequences, there was a difference between our system results and the largest RNAdistance results. However, for these three sequences, we argue that our results presented mutated structures with less similarity to the original structures, when comparing to the structures with the largest RNAdistance. Thus, overall our system outperformed RNAdistance results in at least some of the cases.

The distance measure presented in this article, DoPloCompare, has several advantages with respect to previously suggested techniques (most commonly used are the ones described in [6]):

• The measure is used with the dot plot representation, whereas to the best of our knowledge no other measure was suggested beforehand for this type of representation. Probability and energy dot plots have an increased potential to be used even more in the future, in cases where a more sophisticated analysis is needed besides inspecting the predicted secondary structures. The measure is inversely proportional to the similarity (or proportional to the dissimilarity) between the structures being compared.

• The metric combines coarse and fine-grain characteristics, provided by the distance measure and the correlation respectively, and thus balances both the distance between the nucleotides and the structural elements (e.g., hairpin, loop, etc.) in the compared structures.

• DoPloCompare is easily tuned with regard to the distance function (Hausdorff, RMS, etc.), the correlation algorithm (histograms correlations, traditional correlation, etc.) and their combination.

• DoPloCompare can receive the structures as input from a list of popular folding programs' output files, such as Mfold and the Vienna RNA package.

• DoPloCompare can be easily incorporated into two applications as illustrated here: the RNA design, and RNA conformational rearranging mutation prediction. It provides some good results in comparison to known methods.

There are a number of avenues that are possible to pursue in the future for the extension of DoPloCompare and the presented application:

• DoPloCompare: operation on more sophisticated dot plots that contain more information (e.g., probability and/or energy values). Our technique using histogram correlation and RMS distance permits for potential extensions that will utilize numerical values contained within dots, much like in the case of digital images.

• DoPloCompare: integrate into the RNAMute mutation analysis tool [16], which might be found beneficial in cases where dot plots are dealt with for visualization.

Based on our experiments in comparing DoPloCompare to the traditional distance measures available in RNAdistance, it should be noted that we still recommend utilizing the latter as default unless exceptional cases are dealt with.

## Conclusion

We have provided a new technique borrowed from image processing that utilizes the dot plot representation to compare RNA secondary structures. The technique can be advantageous in some cases and when dot plots are dealt with it can be used as a baseline for other RNA structure based applications.

## Methods

### RNA suboptimal solutions

In order to make folding predictions based on an RNA secondary structure, we used the RNAsubopt [40] available in the Vienna RNA package, a program that predicts all suboptimal secondary structures of a given sequence based on thermodynamics and base pairing rules [3]. Alternatively, we might as well use the suboptimal solutions calculated by Mfold. RNAsubopt, like many other RNA folding approaches, uses a free energy minimization procedure. It is expected that the native fold of the sequence is close to the minimum free energy (mfe) structure. We are interested in all suboptimal solutions because in nature RNA often folds into a suboptimal structure (and also because of limitations of thermodynamic models), which may cause the mfe structure to be different than the native fold. For a given sequence, RNAsubopt calculates all suboptimal secondary structures within an energy range above the minimum free energy. It outputs the suboptimal structures – sorted by mfe – in a dot-bracket notation, followed by the energy in kcals/mol. Originally, a different method for calculating suboptimal solutions was devised by Zuker [41], and is used in Mfold.

### Creating the point mutations

In order to create all the possible single point mutations for a given sequence, we simply traverse along the sequence and for each position i do:

Let *N*_1_, *N*_2 _and *N*_3 _be the three possible nucleotides which are different than the nucleotide in position i. Let *SEQ*(*j*, *k*) denote the subsequence starting in position j in the original sequence and ending at position k (in case *k *<*j *return an empty sequence).

Return:

*SEQ*(1, *i *- 1) ∘ *N*_1 _∘ *SEQ*(*i *+ 1, *m*) ∪

*SEQ*(1, *i *- 1) ∘ *N*_2 _∘ *SEQ*(*i *+ 1, *m*) ∪

*SEQ*(1, *i *- 1) ∘ *N*_3 _∘ *SEQ*(*i *+ 1, *m*)

Where *m *is the original sequence length.

### Dot plot diagrams

A dot plot is a diagram comprised of dots on two axes. Each of the axis represents some sort of data. A dot in location (x, y) represents some measure between the location x in the X-data axis and location y in the Y-data axis. For example, the axis can represent two sentences, and the dots can represent the locations where the sentence on the X-axis and the sentences on the Y-axis contain the same word. In biology, dot plots are often utilized for representing alignments between sequences. Specifically in RNA, a dot plot is often used as an image representation of an optimal base pairing between any two nucleotides in the RNA sequence, based on minimum free energy consideration. Both Mfold [1] and the Vienna RNA package [6] present dot plots as part of their standard outputs, but instead of dots they use squares. Mfold presents dot plot diagrams based on the minimum free energy of the suboptimal folding options of the sequence, where each folding option square is painted with a different color. The Vienna RNA package, on the other hand, presents a different dot plot diagram where each square in the diagram represents the probability of a base pairing in that location in the sequence; the larger the probability, the larger the representing square.

In our approach, we compare each folding option separately, and require a separate dot plot diagram for each suboptimal solution (as opposed to Mfold's dot plot, for example). To comply with this constraint, we created a simplified dot-plot-like matrix with the following properties:

1. Let *LEN *be the length of the sequence being observed, then the matrix is of two dimensions, and of size *LEN *× *LEN*.

2. The matrix cell (i, j) can contain either one of the values {0, 1} where 1 means that i match j in the current folding option and 0 otherwise.

Giving the fact that if nucleotide in position i matches a nucleotide in position j, j will also match i, clearly the matrix is symmetric along the diagonal.

### Histograms

Histograms have been widely and very successfully used in image processing and shape analysis. Although originally they were used to study the data statistics, they have recently been found to be critical for identification, recognition, and distance computations as well, e.g., [42,43]. Histograms constitute the building block of most state of-the-art shape identification and classification systems. Moreover, it has been recently shown that under very general conditions, histograms can uniquely identify a shape with extremely high probability [44]. This provides a very clear motivation to consider histograms for RNA secondary structure analysis, as suggested in this paper.

In order to explain the "Dist" and "Corr" components of Equation (1) in more detail, we will first concentrate on "Corr" (which is, in our case, the Cross Correlate expressed in Equation 4). Next, in the Subsection about the distance between groups of points in the plane, we will concentrate on "Dist" (which is, in our case, the RMS expressed in Equation 5).

In this manuscript we are using normalized cross-correlation between two one-dimensional vectors, in order to determine the level of similarity between these vectors.

Cross correlation is a standard method of estimating the degree to which two vectors are correlated.

Consider two vectors, *X*(*i*) and *Y *(*i*), where *i *= 0, 1, 2...*N *- 1.

The cross correlation *Corr *at delay *d *is defined as:

(3) $$Corr(d)=\frac{\sum_{i}\left[(X(i)-MX)\times (Y(i-d)-MY)\right]}{\sqrt{\sum {\left(X(i)-MX\right)}^{2}\times \sum {\left(Y(i-d)-MY\right)}^{2}}}$$

Where *MX *and *MY *are the means of the corresponding series, and *d *= 0, 1, 2,... *N *- 1 represents all the possible delays.

In this paper we refer to the cross correlation between *X *and *Y *as:

(4) *Cross_Correlate*(*X*, *Y*) = *Max*_*d*_(*Corr*(*d*))

Where *Corr*(*d*) is as defined in Equation 3.

**Figure 10:**
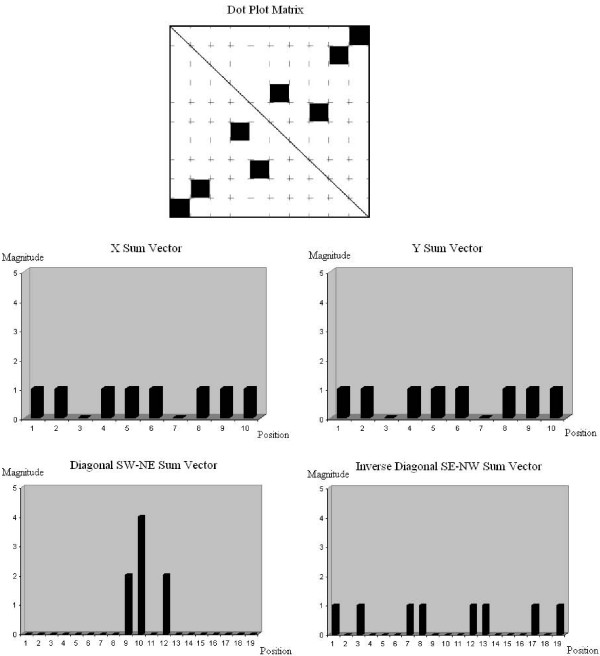
**Sum vectors for dot-plot matrix**. A 10 × 10 dot plot diagram sample, along with its four representing sum vectors: • The 'X Sum Vector' which sums all the dots values along the X axis of the diagram. • The 'Y Sum Vector' which sums all the dots values along the Y axis of the diagram. • The 'Diagonal SW-NE Sum Vector' which sums all the dots along the SW-NE diagonal of the diagram. • The 'Inverse Diagonal SE-NW Sum Vector' which sums all the dots along the SE-NW inverse diagonal of the diagram. Where 'Position' refers to a position along the scanned axis, and 'Magnitude' stands for the summed pixel values at that position. The four vectors are compared to other dot plot diagram's vectors in the process of correlation.

In order to build a one-dimensional series vector from the two-dimensional matrix that represents the original Dot Plot diagram, we traverse the diagram, each time on a specific axis, and sum all the values on that axis (e.g. sum all the columns on the X axis, or sum all the rows on the Y axis). In this manner we obtain a one-dimensional vector for each axis, which can be correlated to the matching axis vector of the second matrix that represents the mutated Dot Plot diagram (see example in Figure 10).

The Cross-Correlation grade will be maximal when the two compared vectors are identical, or contain identical areas. We have used this feature in our assumptions, as explained in the DoPloCompare Section under the formulas explanation Subsection.

### Distance between groups of points in the plane

The matching and analysis of geometric features is an important problem that arises in various computational areas, e.g., computer vision and pattern matching. In general, we are given two sets of points A and B, and we wish to determine how much they resemble each other (for more information see for example [45]). Usually we can apply certain transformation on one of the sets, e.g., translate, scale and/or rotate, in order to be matched with the other set as closely as possible.

In order to measure the level of affinity between two dot plot diagrams, various measure functions have been devised. Two such common measures are the Hausdorff distance [45] and the Root Mean Square distance (RMS) [46-48]. Note that the Hausdorff distance has also been popular in image processing [34]. In this paper we use the RMS measure (see the definition of RMS in Equation 5 given below), but the system can be easily adapted to use the Hausdorff distance or any other measure. No alignment between the groups is performed prior to the measurement, after several trials have shown no difference, for this particular (RMS) distance, if an alignment is added, and therefore the alignment procedure was removed for performance considerations.

The Root Mean Square distance for a set B from set A is:

(5) $$RMS(A,B)=\sqrt{\frac{1}{n}\sum_{a\in A}{\left\|a-N_{B}(a)\right\|}^{2}}$$

Where *n *is the size of group *A *and *N*_*B*_(*a*) is the nearest neighbor of point *a *in group *B*.

The mark ∥ in this context refers to the Euclidean norm.

The measure simply sums and normalizes the distances between each point in A to its nearest neighbor in set B. Clearly, when the two sets lie on top each other, the RMS score will be zero. Alternatively, for sets of different spreading in the plane the RMS distance will increase.

RMS distance between groups of points uses nearest neighbor queries in order to find the point from the other group from which to calculate each point's distance. In order to calculate nearest neighbor queries we implemented a version of planar Voronoi diagram [49], with pre-process time of *O*(*n *log *n*), which answers nearest neighbor queries in *O*(log *n*) for a group of n locations in the plane. We chose not to further discuss standard Voronoi diagram since its implementation and use have no influence on the system output but only on the algorithm run-time.

In our approach, we look for the distance between groups of dots in the base pairing plane, i.e., we look for the RMS distance between two dot plot diagrams as described in the "DoPloCompare" Section.

### Base-pairing distance

As a baseline method for comparing two secondary structures we used RNAdistance, which is part of the Vienna RNA package. It reads RNA secondary structures and calculates a "base-pair distance" given by the number of base pairs present in one structure-but not the other. We use this method as a measure of success in identifying the largest distance between the original sequence and the mutated sequence. We compare our results to the RNAdistance fine-grain method where two structures in dot-bracket notations are being compared. The coarse-grain method was also considered, however it provides poor results and therefore it was discarded.

### RNA-design

We use for illustration the RNAinverse as an RNA-design bioinformatics method that is part of the Vienna RNA package. It searches for sequences folding into a predefined structure, thereby contituting an inverse folding algorithm. For each search the output includes both the best sequence that was found and its Hamming distance to the start sequence. If the the search was unsuccessful (i.e., the structure is undesignable), a structural distance to the target by using the standard base pairing distance is added to the output. We have replaced the base pairing distance with our DoPloCompare distance in RNAinverse, and compared our results to the original RNAinverse before the replacement.

## Competing interests

The authors declare that they have no competing interests.

## Authors' contributions

TI and SM and AA worked on the software design, carried our development and implementation, and participated in drafting the manuscript. DB and GS conceived the study, coordinated the software design and drafted the manuscript.

## Supplementary Material

Additional file 1 **Dataset of ribosomal RNA fragments**. A supplementary file containing a dataset of ribosomal RNA fragments of *thermus thermophilus *HB8. The dataset is based on the experiment described in [39]. It contains 21 fragments that are used for testing the new method introduced here for measuring distances between RNA secondary structures and comparing it with traditional methods. Click here for file
