# Supplementary material for: An image processing approach to computing distances between RNA secondary structures dot plots
Source: Algorithms Mol Biol. 2009 Feb 9;4:4. doi: 10.1186/1748-7188-4-4 (PMC2677394; doi:10.1186/1748-7188-4-4)
Supplement: Additional file 1 — Dataset of ribosomal RNA fragments. A supplementary file containing a dataset of ribosomal RNA fragments of thermus thermophilus HB8. The dataset is based on the experiment described in [39]. It contains 21 fragments that are used for testing the new method introduced here for measuring distances between RNA secondary structures and comparing it with traditional methods. [file 1748-7188-4-4-S1.pdf]

An Image Processing Approach to Computing Distances Between RNA Secondary Structures Dot Plots

Supplementary Data

The following Dataset was used in the Results section of the article:

- Dataset of the Ribosomal RNA fragments of *Thermus thermophilus HB8* based on [1] containing the following 21 fragments:

>Entry:A_(765-816) Length:52 Origin:rRNA of the *Thermus thermophilus* [AC:NC_006461]
gaaagcguggggagcaaaccggauuagauacccgguaguccacgcccuaaa

>Entry:E_(68) Length:46 Origin:rRNA of the *Thermus thermophilus* [AC:NC_006461]
ccggaaggucaaggggaggggugcaagccccgaaccgaagccccgg

>Entry:A_(1241-1296) Length:56 Origin:rRNA of the *Thermus thermophilus* [AC:NC_006461]
gcccacuacaaagcgaugccacccggcaacggggagcuaaucgcaaaaaggugggc

>Entry:A_(820-879) Length:53 Origin:rRNA of the *Thermus thermophilus* [AC:NC_006461]
gcgcgcuaggucucugggucuccugggggccgaagcuaacgcguuaagcgcgc

>Entry:A_(588-651) Length:64 Origin:rRNA of the *Thermus thermophilus* [AC:NC_006461]
gccuggggcgucccaugugaaagaccacggcucaaccgugggggagcgugggauacgcucaggc

>Entry:A_(995-1045) Length:55 Origin:rRNA of the *Thermus thermophilus* [AC:NC_006461]
augcuagggaacccgggugaaagccuggggugccccgcgaggggagcccuagcac

>Entry:B_(1052-1107) Length:56 Origin:rRNA of the *Thermus thermophilus* [AC:NC_006461]
ccaggagguuggcuuagaagcagccauccuuuaaagagugcguaauagcucacugg

>Entry:B_(589-668) Length:82 Origin:rRNA of the *Thermus thermophilus* [AC:NC_006461]
cacggucguggggcgagcuuaagccguugaggcggaggcguagggaaaccgaguccgaacagggcgucuaguccgcggccgug

>Entry:A_(136-227) Length:93 Origin:rRNA of the *Thermus thermophilus* [AC:NC_006461]
ccggaagagggggacaacccggggaaacucgggcuaaucccccauguggacccgccccuuggggugugucccaaagggcuuug
cccgcuuccgg

>Entry:A_(1113-1187) Length:74 Origin:rRNA of the *Thermus thermophilus* [AC:NC_006461]
ccccgccguuaguugccagcgguucggccgggcacucuaacgggacugcccgcgaaagcgggaggaaggagggg

>Entry:B_(865-911) Length:46 Origin:rRNA of the *Thermus thermophilus* [AC:NC_006461]
cacugauagggcuagggggcccaccagccuaccaaacccugucaaa

>Entry:E_(2676-2731) Length:57 Origin:rRNA of the *Thermus thermophilus* [AC:NC_006461]
cgcaccucugguuucccagcugucccuccaggggcagaagcuggguagccaugugcg

>Entry:E_(99,100,101) Length:79 Origin:rRNA of the *Thermus thermophilus* [AC:NC_006461]
ggacccgggaagaccacccgguggaugggccgggggguguaagcgccgcgaggcguugagccgaccggucccaaucgucc

>Entry:E_(90,91,92) Length:76 Origin:rRNA of the *Thermus thermophilus* [AC:NC_006461]
cggcucgucgcauccuggggcugaagaaggucccaaggguugggcuguucgcccauuaaagcggcacgcgagcugg

>Entry:E_(89) Length:43 Origin:rRNA of the *Thermus thermophilus* [AC:NC_006461]
ggcugaucucccccgagcguccacagcggcggggagguuuggc

>Entry:D_(8,9,10) Length:53 Origin:rRNA of the *Thermus thermophilus* [AC:NC_006461]
aaugggggaacccggccggcgggaacgccggucaccgcgcuuuugcgcggggg

>Entry:A_(1420-1480) Length:56 Origin:rRNA of the *Thermus thermophilus* [AC:NC_006461]
cgggcucuacccgaagucgccgggagccuacgggcaggcgccgaggguagggcccg

>Entry:A_(240-286) Length:47 Origin:rRNA of the *Thermus thermophilus* [AC:NC_006461]
cccaucagcuaguugguggguaauggcccaccaaggcgacgacggg

>Entry:A_(442-492) Length:41 Origin:rRNA of the *Thermus thermophilus* [AC:NC_006461]
cccgggacgaaacccccgacgaggggacugacgguaccggg

>Entry:E_(65,66) Length:57 Origin:rRNA of the *Thermus thermophilus* [AC:NC_006461]
acuguuuaccaaaaacacagcucucugcgaacucguaagaggagguauagggagcga

>Entry:E_(86,87) Length:39 Origin:rRNA of the *Thermus thermophilus* [AC:NC_006461]
gacugcgaggccugcaagccgagcaggggcgaaagccgg

Bibliography

1. Yusupov MM, Yusupova GZ, Baucom A, Lieberman K, Earnest TN, Cate JH, Noller HF: Crystal structure of the ribosome at 5.5 A resolution. Science 2001, 292:883–896.
